# Bioinformatics in crop research: using genomic data for crop improvement

**DOI:** 10.3389/fpls.2026.1868979

**Published:** 2026-08-19

**Authors:** Muhammad Shahid Iqbal, Zareen Sarfraz, Muhammad Mujahid, Yusra Zarlashat, Alia Ambreen, Yasmeen Siddiqui, Amjad Ali, Rashid Abbas Khan, Zeeshan Ahmad, Muhammad Sajid Iqbal, Abdul Waheed, Asgar Ali

**Affiliations:** 1 Agricultural Genomics Institute at Shenzhen, Chinese Academy of Agricultural Sciences, Guangdong, China; 2 Department of Biochemistry, Government College University Faisalabad, Faisalabad, Pakistan; 3. School of Environmental Science, Liaoning University, Shenyang, China; 4 Department of Biological Sciences, College of Science, King Faisal University, Al Hofuf, Al Ahsa, Saudi Arabia; 5 Department of Plant Protection, Faculty of Agricultural Sciences and Technologies, Sivas University of Science and Technology, Sivas, Türkiye; 6 College of Graduate Studies, University of South Africa, Pretoria, South Africa; 7 Department of Biosciences, COMSATS University Islamabad, Islamabad, Pakistan; 8 Engineering Research Center of Biomass Materials, Ministry of Education, School of Life Science and Engineering, Southwest University of Science and Technology, Mianyang, China; 9 Department of Integrative Agriculture, College of Agriculture and Veterinary Medicine, United Arab Emirates University, Al Ain, United Arab Emirates

**Keywords:** bioinformatics, crop improvement, genetic engineering, genomics, molecular breeding, omics integration (multi-omics), sustainable agriculture

## Abstract

Sustainable crop development aims to maintain or increase yields while reducing environmental impact and managing the challenges imposed by climate change. As the global population grows and arable land becomes scarcer, the integration of molecular breeding with bioinformatics has emerged as an effective strategy for long-term crop improvement. Bioinformatics enables researchers to analyze and interpret the vast quantities of genetic data generated by high-throughput sequencing, making it possible to identify molecular markers, candidate genes, and regulatory networks linked to specific agronomic traits, which breeders then translate into focused, ecologically sustainable breeding programs. This approach has enabled major progress across several fronts: the identification of genes conferring resistance to biotic stressors (pests, pathogens) and abiotic stressors (drought, salinity, heat); the development of nutrient-efficient, low-input crop varieties; the improvement of agronomic performance and nutritional quality through identification of yield- and quality-related genes; and the conservation and deployment of genetic diversity to safeguard long-term breeding sustainability. By combining genomic data with precision breeding techniques, researchers are developing crops that are better adapted to a growing population and a changing climate, positioning the integration of molecular breeding and bioinformatics as a central pillar of future global food security.

## Graphical Abstract

**Figure:**
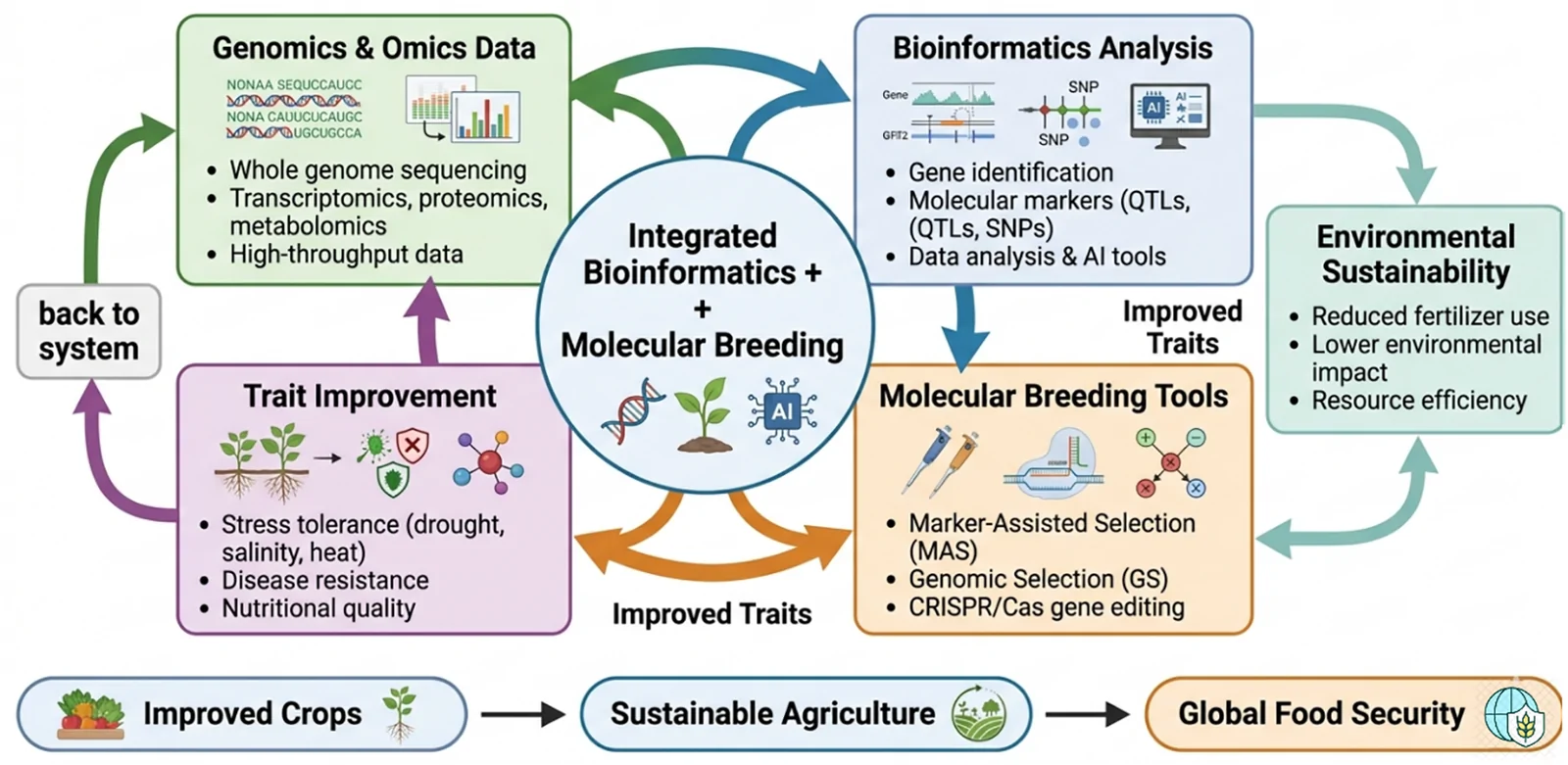

## 1 Introduction

Agricultural systems will be under extreme pressure to provide enough food while reducing their negative effects on the environment, with a projected 9.7 billion people on the planet by 2050 (Webb et al., 2020). More frequent droughts, floods, and very high temperatures are some of the problems imposed by climate change that may have a detrimental effect on agricultural yields and quality. Traditional breeding techniques were effective in the past, but are not able to meet these quickly changing needs (Ahmad et al., 2024). Creating crop types that can flourish in these harsh environments while preserving high yields and nutritional quality is the main goal of sustainable agricultural revolution (Albahri et al., 2023).

Plant genome sequencing has become less expensive and time-consuming because to the quick development of DNA sequencing technology, which has led to the discovery of genomic data. Databases and tools for bioinformatics are of utmost importance to manage, evaluate, and interpret this enormous volume of data, which allows scientists to find genes and molecular indicators linked to possessing favorable qualities (Siddiqui, 2024). These features are subsequently included into top crop varieties using molecular breeding methods i.e., marker-assisted selection (MAS), which significantly improves the breeding process. Molecular breeding and bioinformatics together have the potential to completely transform agricultural improvement through providing a more focused and effective method of creating strong and productive crop types (Chavhan et al., 2024).

## 2 Molecular breeding techniques

DNA markers are used in molecular breeding methods, including genomic selection (GS), quantitative trait loci (QTL) mapping, and MAS to find plants with desired traits (Kumar et al., 2024). These markers are different DNA sequences linked to certain genes or genomic areas that affect how a desired trait, including yield, disease resistance, or drought tolerance, is expressed (Younis et al., 2020). Breeders save time and money by using these markers to choose plants with the desired characteristics without performing any field tests. Further enhancing crop performance, which is possible by using molecular breeding methods to pyramid many advantageous characteristics into a single variety (Ahmar et al., 2020).

### 2.1 Genomic selection

**Figure 1:**
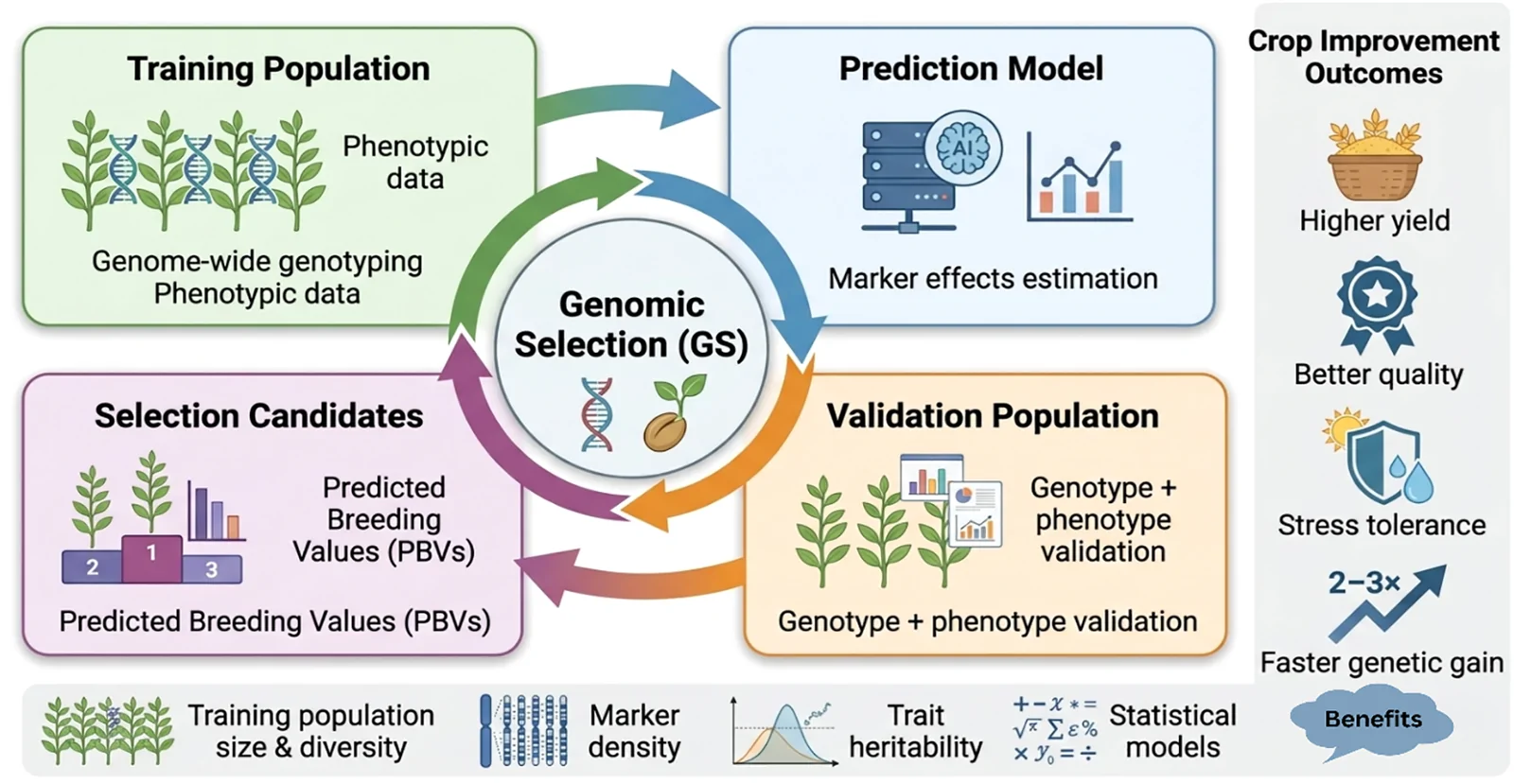
Systematic workflow of genomic selection (GS) for accelerated crop improvement.

A more contemporary molecular breeding technique called GS uses genome-wide markers to predict breeding value (Anilkumar et al., 2023). Four steps are involved in the process (**Figure 1**): firstly, the training population is genotyped using genome-wide markers; secondly, a robust prediction model based on marker effects is developed; thirdly, an independent set of genotyped and phenotyped individuals is used for thorough validation; and lastly, the validated model is applied for predicting breeding values for new selection candidates (Sehrawat et al., 2023).

Numerous crop development programs have shown notable success using genomic selection. The use of GS in maize breeding programs has produced impressive results; research has shown that genetic gains rise up to three times when compared to traditional breeding techniques (Voss-Fels et al., 2019). GS has been effectively used by wheat improvement programs to increase grain yield and quality attributes, with prediction accuracies ranging from 0.4 to 0.8 across various traits and populations (Subedi et al., 2023). Researchers have found that GS implementation has been very successful in soybean breeding, with prediction accuracies for a variety of variables, including as yield and oil content, reaching 0.92 (Stewart-Brown et al., 2019) (**Table 1**). These include of the training population’s size and genetic diversity, marker density, trait heritability, and distribution across the genome, and the choice of suitable statistical models for predictions.

**Table 1:** Comparison of statistical and machine-learning models used for genomic selection.

Statistical model	Key assumption	Best suited for	Trade-off	Reference
GBLUP	Infinitesimal model; all markers have small, equal, normally-distributed effects	Polygenic traits; routine, large-scale genomic selection	Computationally efficient; underperforms when a few loci have large effects	VanRaden, 2008
Bayesian Ridge Regression	Similar to GBLUP but allows marker-specific shrinkage	Traits with a mix of small and moderate marker effects	More flexible than GBLUP; higher computational cost	Meuwissen et al., 2001
BayesA/BayesB/BayesC	Marker-specific variances; some markers can have disproportionately large effects	Oligogenic traits with a few moderate-to-large-effect loci	Better capture of major loci; higher computational demand and risk of overfitting with small training sets	Meuwissen et al., 2001
RKHS	Non-parametric; captures non-additive (epistatic) marker interactions via a kernel matrix	Traits with suspected epistasis or non-additive gene action	Flexible; less interpretable, kernel choice affects performance	Gianola and van Kaam, 2008
LASSO	Linear model with L1 penalty; shrinks small marker effects to zero	Oligogenic traits where variable selection aids interpretation	Improves interpretability; can be unstable with highly correlated markers	Sarkar et al., 2015
Random Forest	Ensemble of decision trees; no explicit genetic model assumed	Complex traits with non-linear marker interactions, large datasets	Captures non-linearity; low interpretability, high computational cost	Sarkar et al., 2015

The predictive power of genomic selection depends on the statistical model used to relate genome-wide marker genotypes to phenotypic performance, and several frameworks are now routinely compared in crop breeding programs (Meuwissen et al., 2001). Best Linear Unbiased Prediction (BLUP) and its genomic extension, GBLUP, estimate breeding values assuming an infinitesimal model in which all markers contribute small, normally distributed effects, and remain the most widely used approach because of their computational efficiency and robustness across traits (VanRaden, 2008). Bayesian frameworks, including Bayesian Ridge Regression, BayesA, BayesB, and BayesC, relax this assumption by allowing marker-specific variances, so that a subset of markers can have disproportionately large effects; they tend to outperform GBLUP for traits influenced by a few moderate- to large-effect loci but are more computationally intensive (Meuwissen et al., 2001). Reproducing Kernel Hilbert Space (RKHS) regression captures non-additive (epistatic) effects through a kernel-based similarity matrix and is useful when trait architecture is not well approximated by additive models (Gianola and van Kaam, 2008). More recently, machine-learning approaches such as LASSO regression and Random Forests have been applied to genomic prediction; LASSO performs variable selection by shrinking small marker effects to zero, which can improve interpretability for oligogenic traits, while Random Forests capture non-linear marker interactions without requiring an explicit genetic model, at the cost of reduced interpretability and higher computational demand, and have been reported to outperform LASSO and ridge regression in some genomic prediction comparisons (Sarkar et al., 2015). No single model consistently outperforms the others across all traits and populations, so model choice in practice is guided by trait genetic architecture (polygenic versus oligogenic), training population size, and computational resources, and cross-validation within the target breeding population remains the standard way to select the best-performing model (de los Campos et al., 2013). Reported prediction accuracies vary accordingly by crop and trait: beyond the wheat, maize, and soybean examples above, GS has achieved prediction accuracies of approximately 0.3-0.6 for grain yield and quality traits in rice, and 0.4-0.7 for malting quality and agronomic traits in barley, reflecting differences in training population size, marker density, and trait heritability across these species (Rooney et al., 2023).

The figure illustrates the integration of genotypic and phenotypic data to develop high-precision AI prediction models within a circular breeding cycle. By utilizing Predicted Breeding Values (PBVs), the system enables the rapid identification of superior selection candidates, resulting in a faster genetic gain and the development of high-yielding, climate-resilient cultivars (**Table 2**).

**Table 2:** Important crops and the modern breeding approaches being used to improve important agronomic traits.

Crop	Trait	Approach	Reference
Wheat	Grain yield	Genomic selection	(Kumar et al., 2024)
Tomato	Fruit quality	Genomic selection, MAS	(Rajendra, 2025)
Maize	Drought tolerance	GWAS, MAS	(Wang et al., 2025a)
Rice	Grain size and quality	GWAS, gene editing	(Hori and Sun, 2022)
Soybean	Disease resistance	GWAS, MAS	(Wang et al., 2025b)
Barley	Malting quality	GWAS, Genomic selection	(Rooney et al., 2023)
Potato	Tuber yield and disease resistance	Gene editing, MAS	(Sapakhova et al., 2025)
Cassava	Virus resistance	Gene editing, MAS	(Huang et al., 2024)
Cotton	Fiber quality and herbicide tolerance	Genomic selection, MAS	(Zeng et al., 2023)
Canola	Oil content and composition	GWAS, Genomic selection	(Hu et al., 2022)
Grapes	Disease resistance and flavor	GWAS, Gene editing	(Sun et al., 2023)

### 2.2 MAS

**Figure 2:**
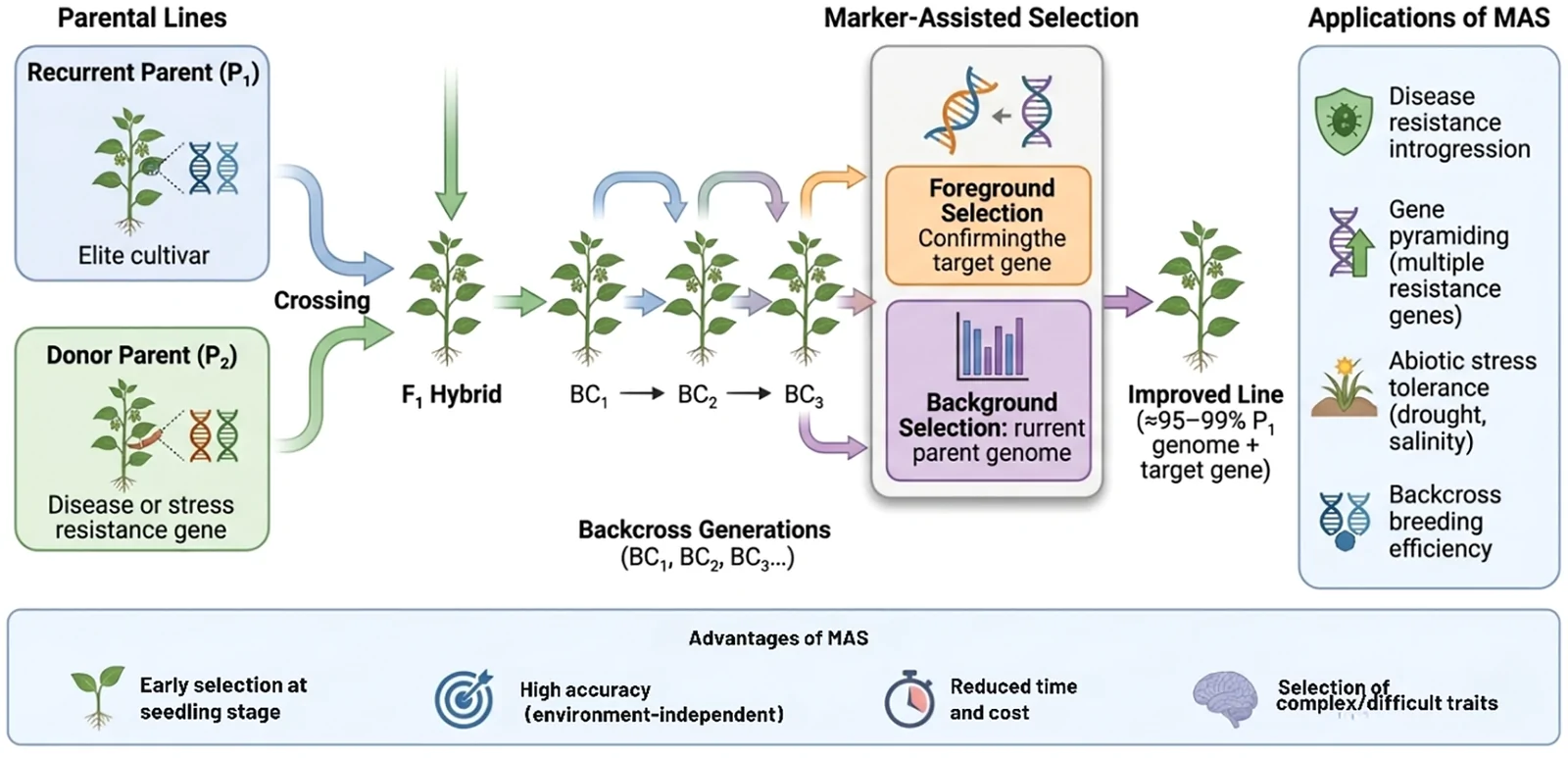
Marker-assisted selection (MAS) for gene introgression and background genome recovery.

MAS is predicated on the idea of genetic linkage, which states that people with the desired qualities may be chosen using DNA markers that are closely associated with the genes regulating those traits (**Figure 2**). This method eliminates the need for in-depth phenotypic analyses and enables the early and effective selection of plants with the necessary traits (Cobb et al., 2019). MAS has been used extensively in crop development programs for many reasons, including, disease-resistant genes from wild relatives being introduced into top cultivars (Khalid et al., 2025). Multiple resistance genes are pyramided to increase durability (Mhetre et al., 2025). Backcross breeding preserves the ideal background of the recurrent parent while transferring certain features (Begna, 2021). Early selection favor features that can withstand abiotic stressors, such as resistance to salt and drought (Raj and Nadarajah, 2022). MAS is better than traditional phenotypic selection in a number of ways. Greater selection efficiency and accuracy since environmental influences do not affect DNA markers (Lema, 2018). Saved time and money on field assessments since selection can be done at the seedling stage (Lema, 2018). Capacity to choose for qualities like disease resistance or root properties that are costly or difficult to characterize (Sunilkumar et al., 2023).

A donor parent (P_2_) carrying a desirable gene or quantitative trait locus (QTL), such as disease or abiotic stress resistance, is crossed with an elite recurrent parent (P_1_). In each backcross generation (BC_1_, BC_2_, …), plants are screened using foreground DNA markers to confirm the presence of the target gene, and background markers to select individuals with the highest proportion of the recurrent parent genome, thereby minimizing linkage drag. Iterative marker-assisted backcrossing rapidly restores the elite P_1_ background (≈95–99%) while retaining the introgressed target gene from P_2_. MAS accelerates breeding by enabling early selection, increasing accuracy, and reducing dependency on field phenotyping, particularly for traits that are costly or difficult to measure.

### 2.3 CRISPR/Cas for gene manipulation

**Figure 3:**
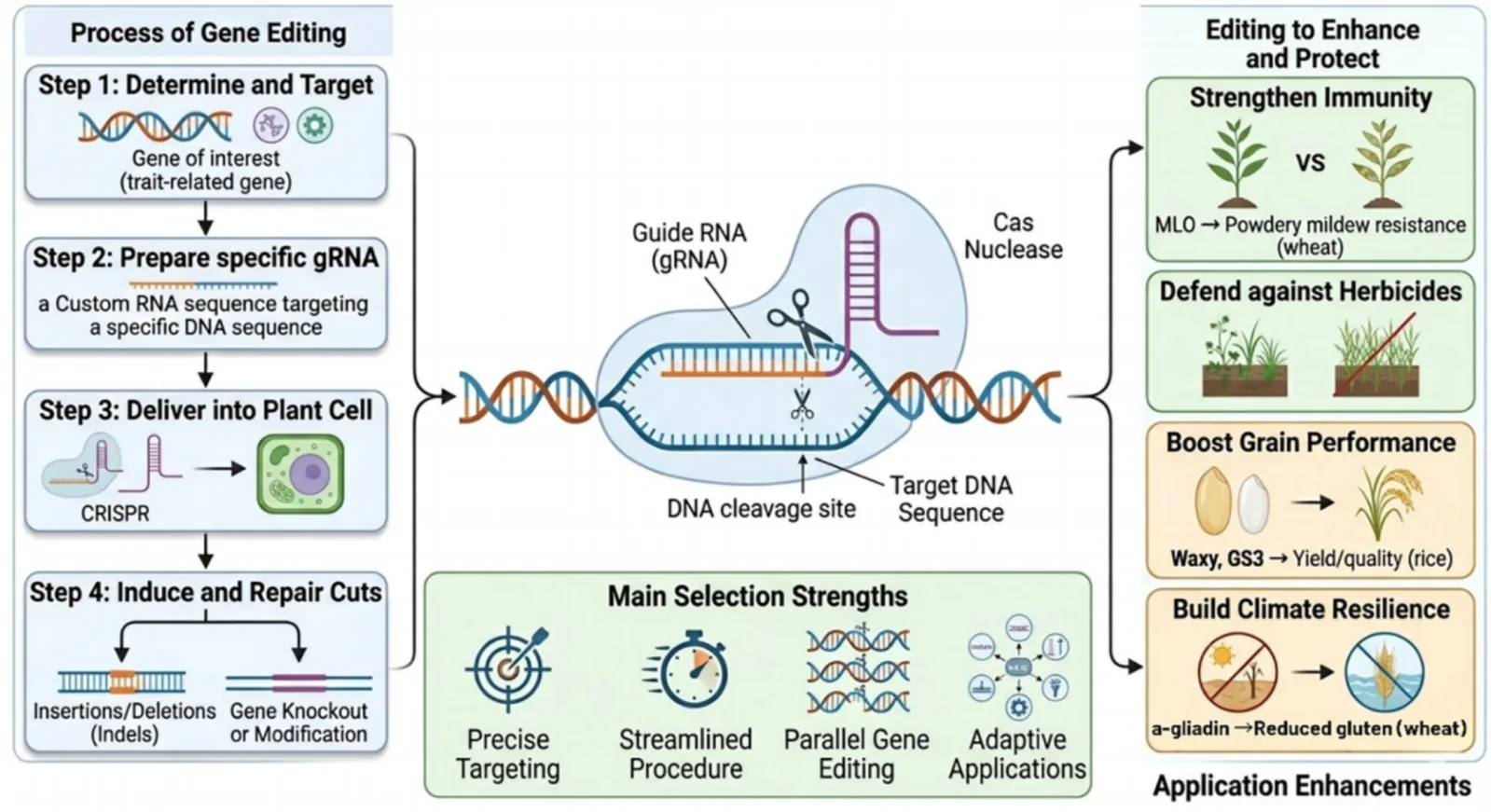
Overview of the CRISPR/Cas gene-editing process and its strategic applications in crop improvement.

Crop improvement has greatly benefited from genome editing technologies, especially the CRISPR/Cas (Clustered Regularly Interspaced Short Palindromic Repeats/CRISPR-associated protein) system. These systems combine a programmable nuclease (Cas) with a guide RNA that precisely targets particular genomic regions (Alamillo et al., 2023) (**Figure 3**). They are derived from bacterial and archaeal adaptive immune mechanisms. Researchers may now accomplish targeted genetic alterations, such as mutations, insertions, and deletions at specific genomic sites, due to the precise construction of guide RNAs. This ability makes it possible to quickly and precisely alter genes that regulate crucial agronomic characteristics (Ruden, 2025).

CRISPR/Cas systems have several benefits over conventional transgenic methods when it comes to improving crops. These systems are divided in four crucial domains: accuracy, effectiveness, multiplexing capacity, and adaptability (Sun et al., 2024; Sarfraz et al., 2025a). Targeted gene editing is made possible by the accuracy of CRISPR/Cas without random DNA integration. The technology is more effective to cause mutations than earlier techniques like TALENs and zinc-finger nucleases (Ahmadikhah et al., 2025). Its multiplexing capacity makes the simultaneous editing of many genes possible by designing many guide RNAs. Moreover, CRISPR/Cas’s adaptability enables a range of uses, including transcriptional regulation and gene knockouts (Devillars et al., 2024). CRISPR/Cas technology has been successfully used by agricultural researchers to improve grain quality, herbicide tolerance, and disease resistance in a variety of crop species (Dong et al., 2021). Notable achievements include producing wheat lines with reduced immunogenic gluten content through CRISPR/Cas9 editing of alpha-gliadin genes (Sanchez-Leon et al., 2018; Wani et al., 2020), engineering glutinous, reduced-amylose rice by editing the Waxy gene (Zhang et al., 2018), improving grain size, yield, and quality in hybrid rice through GS3 editing (Huang et al., 2022), and conferring heritable resistance to powdery mildew in wheat by simultaneously knocking out all three TaMLO homoeoalleles (Wang et al., 2014) (**Table 3**).

**Table 3:** Representative crops improved through CRISPR/Cas genome editing.

Crop	Trait improved	Gene(s)/target	Reference
Wheat	Reduced immunogenic gluten content	alpha-gliadin genes	Sanchez-Leon et al., 2018
Wheat	Powdery mildew resistance	TaMLO-A1/B1/D1	Wang et al., 2014
Rice	Reduced amylose/glutinous grain quality	Waxy	Zhang et al., 2018
Rice	Grain size, yield, and grain quality	GS3	Huang et al., 2022
Potato	Agronomic and quality-trait improvement (CRISPR toolkit review)	Multiple targets	Sapakhova et al., 2025
Cassava	Virus resistance	Multiple targets	Huang et al., 2024

The workflow (left) illustrates the four-step procedure from gRNA preparation to the induction of gene knockouts or modifications. The central mechanism shows the Cas Nuclease creating site-specific cleavage to enable precise targeting. These edits result in significant application enhancements (right), including powdery mildew resistance in wheat, herbicide defense, improved grain quality in rice, and enhanced climate resilience through gluten reduction.

### 2.4 Comparative perspective: GS, MAS, and CRISPR/Cas

Although genomic selection, marker-assisted selection, and CRISPR/Cas editing are often presented as a continuum of molecular tools, they differ substantially in the biological and resource conditions under which each is preferred, and a genuine comparison clarifies their complementary rather than competing roles (Bernardo, 2008). MAS is most efficient for traits controlled by one or a few major-effect loci with well-validated markers (e.g., single-gene disease resistance), where genotyping cost is low and selection can be performed at the seedling stage (Collard and Mackill, 2008); its main limitation is that it captures little of the variance underlying polygenic traits such as yield or drought tolerance, where individual marker effects are small and numerous (Bernardo, 2008). GS overcomes this limitation by using genome-wide markers to capture both major- and minor-effect loci simultaneously, making it the preferred approach for complex, quantitative traits, but it requires a genotyped and phenotyped training population, is more computationally demanding, and its prediction accuracy depends heavily on the genetic relatedness between the training and breeding populations (Meuwissen et al., 2001; de los Campos et al., 2013). CRISPR/Cas, in turn, is not a selection method but a precision-editing tool: it is preferred when the causal gene and its functional variant are already known and a specific, targeted change (knockout, base edit, or allele replacement) is desired, offering far higher precision than MAS-based introgression, which transfers large genomic segments and carries a risk of linkage drag (Scheben et al., 2017). The trade-off is that CRISPR/Cas requires an efficient transformation and regeneration system for the target species, well-characterized guide RNA targets, and, in many jurisdictions, navigation of a distinct regulatory pathway, whereas MAS and GS work within conventional breeding and cross-pollination frameworks (Scheben et al., 2017). In practice, the three approaches are increasingly combined: GS is used to enrich breeding populations for polygenic performance, MAS is used to track and pyramid specific resistance alleles within those populations, and CRISPR/Cas is used to introduce or correct specific alleles that are absent from the available germplasm, so that the choice of method depends on trait architecture, the availability of a validated training population or guide RNA target, and the regulatory and infrastructural context of the breeding program (Scheben et al., 2017).

## 3 Bioinformatics tools

### 3.1 Genomic database

Genomic databases offer crucial sequence information for both crops and their wild relatives serve as the basis for existing molecular breeding. Global sequence repositories including GenBank, ENA, and DDBJ, which keep considerable collections of nucleotide sequences and protein translations, are among these resources (Selvakumar et al., 2024). Furthermore, specialized genomic resources such as comprehensive data on genomes, markers, and QTLs are available through crop-specific databases such as SoyBase, Gramene, MaizeGDB, and Wheat@URGI (Mao et al., 2016).

### 3.2 Bioinformatics tools for sequence analysis and annotation

Detailed sequence analysis for molecular breeding applications is made easier by current bioinformatics technologies. These tools fall into several important functional categories. Advanced alignment tools such as BLAST, ClustalW, and HMMER enable researchers to perform comprehensive sequence comparisons and domain analyses across genomic databases. These methods are essential to identify functional components and conserved areas in crop genomes (Gupta et al., 2021). The identification and characterization of genetic elements are supported by computational tools. Protein-coding gene prediction is possible using AUGUSTUS, while protein family categorization and domain prediction are made easier with InterProScan (Woldesemayat et al., 2017). Blast2GO uses Gene Ontology word assignment and sequence similarity analysis to improve functional annotation (Wimalanathan and Lawrence-Dill, 2021).

The development of molecular markers is an essential part of current crop breeding initiatives (**Table 4**). The most significant matters are SNPs and Simple Sequence Repeats (SSRs) are the main marker systems used in crop improvement. SNPs give benefits for high-throughput genomic investigation, but SSRs offer high polymorphism rates and PCR compatibility (Sinha et al., 2023). Careful consideration of density, dispersion, and repeatability across target populations is necessary for effective marker systems. Validation in particular germplasm collections guarantees dependable use in breeding initiatives (Ray and Ghosh, 2025). A complete understanding of crop biology and trait expression is obtained by combining several omics techniques. This combination consists of transcriptomics analysis in which candidate gene and regulatory network discovery is made possible by RNA sequencing and expression profiling. Network inference, differential expression analysis, and data processing are all supported by contemporary analytical techniques (Tyagi et al., 2022). Metabolomics and proteomics where methods use protein and metabolite studies to directly correlate genetics to phenotype. They make it easier to identify biomarkers and describe post-translational changes (Satrio et al., 2024). Integrated platforms and workflow systems that include several tools and databases are essential to modern breeding processes. These systems consist of the following; For bioinformatics analysis, platforms including Galaxy, R/Bioconductor, and Python-based tools offer complete environments. Both bespoke analytical techniques and standardized procedures are supported by these systems (Johnson et al., 2021).

**Table 4:** Major molecular marker types used in genetic and genomic analysis.

Marker type	Characteristics	Applications	Reference
RFLPs	Low throughput, co-dominant	Diversity analysis, genetic mapping	(Panchariya et al., 2024)
SNPs	High-throughput, abundant, co-dominant	GWAS, MAS, genomic selection	(Susmitha et al., 2023)
AFLPs	High throughput, dominant	Genetic mapping, diversity analysis	(Bunjkar et al., 2024)
SSRs	Co-dominant, highly polymorphic, PCR-based	Diversity analysis, genetic mapping, MAS	(Kumar et al., 2022)
InDels	Co-dominant, PCR-based	Genetic mapping, MAS	(Arabzai and Gul, 2021)
SCARs	Highly reproducible, locus-specific, converted from anonymous markers (like AFLPs)	Marker-assisted selection, germplasm screening, diagnostic assays	(Sinha et al., 2023)
DArT (Diversity Arrays Technology)	High throughput, co-dominant or dominant	Genetic mapping, diversity analysis	(Sinha et al., 2023)
RAPDs	Low reproducibility, dominant, low cost, uses arbitrary primers	Quick genetic diversity assessment, preliminary genetic mapping, fingerprinting	(Biswas and Kumar, 2023)

While transcriptomics, proteomics, and metabolomics are each individually informative, the greatest analytical power comes from integrating two or more omics layers to move from statistical association to functional, mechanistic understanding of a trait (Yang et al., 2021). Several crop-trait-tool combinations illustrate this in practice. In rice, integration of transcriptomic and metabolomic profiling has been used to dissect grain-quality pathways, linking differentially expressed genes to the metabolite changes that determine grain yield and quality traits, with network-inference tools such as weighted gene co-expression network analysis (WGCNA) used to reconstruct the underlying regulatory modules (Zhou et al., 2022). In maize, combining GWAS-derived candidate loci with transcriptome data under drought stress has helped prioritize candidate drought-tolerance genes for functional validation, narrowing large marker-trait association intervals down to a manageable list of regulatory candidates (Wang et al., 2025a). In soybean, proteomic and transcriptomic integration has been applied to characterize the molecular basis of resistance to Rhizoctonia solani root rot, cross-validating defense-related transcripts against their corresponding protein-level changes (Wang et al., 2025b). In grapevine, combined GWAS and metabolomic profiling has linked candidate loci to norisoprenoid biosynthesis, connecting genetic variation directly to a sensory and quality trait (Sun et al., 2023). These examples show that multi-omics integration is most powerful when a genetics-based layer (GWAS or QTL mapping) is used to generate candidate loci, and a molecular-phenotype layer (transcriptomics, proteomics, or metabolomics) is used to functionally validate and prioritize those candidates, a strategy that is increasingly implemented through the integrated platforms (Galaxy, R/Bioconductor, Python-based pipelines) described above.

## 4 Advances in crop improvement

### 4.1 Breeding and engineering of stress-resilient varieties

The development of crop varieties that are resistant to stress has been transformed by the combination of molecular breeding and bioinformatics (Mu et al., 2022). Researchers have made notable progress regarding abiotic stressors through a variety of methods (Nawaz et al., 2023). While genome-wide association studies have identified SNPs linked to heat tolerance in wheat, transcriptome analysis and QTL mapping have successfully identified candidate genes for drought tolerance in maize (Longmei et al., 2021). Transcriptome analysis has been especially useful in treating salt and nutritional deficits. Researchers developed stress-resistant rice cultivars by identifying differentially expressed genes that provide salt tolerance (Shintani and Bono, 2025).

### 4.2 Biotic stress resistance

Comparative genomics techniques have significantly developed agricultural cultivars resistant to plant diseases (Ali et al., 2022). Among the major achievements are: Transcriptome analysis for the characterization of soybean cyst nematode resistance genes (Jain et al., 2016). GWAS technology has also been used to identify genetic markers for corn earworm resistance in maize. Beyond these two examples, comparative and functional genomics approaches have expanded resistance breeding across several other crop-pathogen systems. In soybean, GWAS has been used to map resistance loci against Rhizoctonia solani root rot (Wang et al., 2025b). In cassava, gene-editing and marker-assisted strategies have been deployed to develop resistance against major mosaic and brown streak viruses (Huang et al., 2024). In wheat, simultaneous CRISPR/TALEN-mediated knockout of the three TaMLO homoeoalleles has conferred durable, broad-spectrum resistance to powdery mildew (Wang et al., 2014), while the pyramiding of multiple resistance genes from wild relatives continues to be used to build broad-spectrum, durable resistance in elite cultivars (Khalid et al., 2025; Mhetre et al., 2025). Beyond resistance breeding, evolutionary genomic approaches in wheat landraces have also revealed the role of chromatin remodeling in shaping adaptive trait variation, offering new avenues for breeding climate-resilient varieties (Sarfraz et al., 2026).

### 4.3 Quality and yield improvement

Significant improvements in crop quality and yield are now easier with modern bioinformatics techniques. GWAS and GS methods are frequently used to predict yield performance in cereals and find SNPs linked to grain yield components (Chavhan et al., 2024) (**Table 1**). These strategies have been especially effective in rice and wheat breeding efforts. The use of genetic engineering and MAS has made it easier to enhance basic crops, increasing their nutritional content by adding more elements like iron and provitamin A (Ray and Ghosh, 2025). These technologies create climate-resilient cultivars with better yield stability in the context of heat stress and drought.

### 4.4 Orphan crops and underused species

Orphan crops and underused species, which are important for food security in developing areas, have received special attention (Kumar and Bhalothia, 2020). Even though these crops have historically lacked substantial genetic resources, continuous efforts are creating vital resources and tools for their advancement. They are effectively using evolutionary and participatory breeding techniques to increase their productivity and adaptability to particular environmental niches (Tadele, 2019) (**Table 5**).

**Table 5:** An essential bioinformatics tools and resources for modern crop improvement programs.

Tool/resource	Description	References
BLAST	BLAST Sequence alignment and homology search	(Chen et al., 2021)
Bioconductor	R packages for analyses	(Almeida-Silva et al., 2023)
Primer3	Primer designing	(Syamsidi et al., 2021)
TASSEL	TASSEL Software for diversity analysis	(Monier et al., 2022)
Galaxy	Galaxy Web-based platform for bioinformatics analyses	(Chappell et al., 2022)
MEGA	Molecular evolutionary genetics analysis	(Tamura et al., 2021)
PLINK	Genome-wide association studies	(Truong et al., 2022)
Ensembl Plants	A centralized portal for accessing and visualizing plant genome data	(Contreras-Moreira et al., 2022)
TPIA	The Plant Integrative Omics Database	(Gao et al., 2024)
KEGG	Kyoto Encyclopedia of Genes and Genomes	(Cai et al., 2021)
Cytoscape	An open-source platform for complex network analysis and visualization	(Ono et al., 2025)
InterPro	A tool for classifying protein sequences into families and predicting domains	(Blum et al., 2025)

## 5 Contribution of bioinformatics to crop enhancement

One of the main goals of plant and crop biologists is to understand and discover the molecular and genetic bases of every biological function in plants (Mukhopadhyay et al., 2024). This knowledge helps in the practical application of plants as biological resources in the creation of new cultivars with higher quality and lower prices. The breeding of new crops and the adaptation of existing crops to the new environment are necessary to guarantee ongoing food production since climate change and population growth are imposing more strain on our capacity to generate enough food (Atlin et al., 2017). Simply, developments in genomics have sped up information acquisition, which has helped in the logical annotation of genes, proteins, and phenotypes. As a result, omics data may now be seen as a crucial tool for improving plants (Yang et al., 2021). The nutritional quality and composition of food crops have improved, and agricultural output for food, feed, and energy has increased as a result of several novel gene-finding methods designed for applications to plant genome sequences (Naqvi et al., 2022). In the context of climate change, this combination of bioinformatics and genomic techniques has the potential to preserve food security by speeding up the development of novel cultivars with higher quality and lower environmental and financial costs (Munaweera et al., 2022).

Crop development has benefited greatly from the discovery of sequence analysis and genomic annotation studies. Whole-genome sequencing of a number of species makes it possible for identifying their organization and understanding their functionality, which benefits human agricultural field (Fuentes‐Pardo and Ruzzante, 2017). Due to the huge advancements in the field of omics, a vast quantity of genomics data from plants is now available, and it is possible to check the functions of many plant genes as well as the variables influencing these genes (Abdullah-Zawawi et al., 2022). Scientists have used this information to develop plant varieties that are resistant to pesticides, herbicides, biotic and abiotic stressors (Nawaz et al., 2023). In the domains of comparative genomics, meta-genomics, and evolutionary genomics, a variety of cutting-edge sequencing technologies that are modifications of previously used pyro-sequencing techniques have given us new chances to address the full genome level. The identification and modification of genes associated with certain phenotypic features as well as genomics breeding through marker-assisted variant selection include the contribution of genomics to agriculture (Tiwari et al., 2022). Efforts aimed at obtaining suitable understanding of related molecular information, such as that resulting from transcriptome, metabolome, and proteome sequencing, are also crucial to more accurately represent a genome’s gene content and primary functions (Satrio et al., 2024). Current developments in bioinformatics and genomics offer chances to speed up crop improvement in the following areas.

The term “gene finding” describes the identification of introns and exons in a DNA sequence. Genome sequencing has benefited from bioinformatics, which has demonstrated its ability to locate genes, perform phylogenetic comparisons, and discover of the gene’s transcription factor binding sites (Ejigu and Jung, 2020). A “quantum leap” in the enhancement of quantitative and qualitative traits in economically significant crops will arise from such an approach to discover critical genes and understand their function (Buzdin et al., 2021). Using computer methods, “comparative genetics” (model and non-model plants) show how agronomically significant genes are arranged in relation to one another. This information is then used for other food crops (Brenton et al., 2016). Species-specific nucleotide sequences are already providing information about phenotypic characteristics, even when based on genome comparisons across the few known model plants (Waldvogel et al., 2020).

“Cheminformatics” for the creation of agrochemicals is based on the study of signal reception and transduction pathways. To choose targets and identify possible compounds that may be employed as herbicides, pesticides, or insecticides, thereby enhancing plant quality and quantity (Li et al., 2022). “Agricultural genomics” contributes to a worldwide understanding of plant and pathogen biology, and its use in agriculture would be advantageous since it would provide a wealth of information to enhance crop phenotype (Mishra et al., 2024). Additionally, whole-genome sequencing in plants enables genetic comparisons at the chromosomal size, revealing conserved genetic regions that can help discover and document homologous genomic sequences in related plant species (Hong et al., 2023).

“Full-length cDNA libraries” are used as clones for genetic engineering to increase agricultural productivity and as primary sequence resources for creating microarray probes. These libraries have been used to detect biological characteristics by comparing target sequences with those of model species (Sharma et al., 2022). “Multiple alignments” offer a way to identify previously unidentified genes and determine the number of genes in gene families. Studying the pattern of gene expression in plants is made easier by the multiple alignment data. An efficient method for examining gene function is “mutant analysis” (Bano et al., 2021). Additionally, extensive libraries of mutant lines are crucial bioresources for accelerating up both forward and reverse genetics. “Gene pyramiding” or “gene stacking” refers to the process of assembling many desirable genes from several parent crops in order to improve traits and create top varieties. It is mostly used to improve elite cultivars that already exist for a few undesirable qualities for which genes with significant beneficial effects have been found (Rajput et al., 2023).

“Genetic markers” created that cover the whole genome may enable the detection of genetic diversity and induced variations as well as the identification of specific genes linked to complex phenotypes through QTL (quantitative trait loci) research (Kushanov et al., 2021). Researchers studying plant-pathogen interactions may now more easily find defense/disease-resistant gene-enzymes with their promoter region and transcription factor that support immunity and defense mechanisms due to in silico genomics technology. Bioinformatics has also made it possible for scientists to enhance the nutritional value of such plants by altering the genome of plants (Shafi et al., 2019). Researchers have successfully inserted genes to raise vitamin A levels in the rice genome. Because genetically modified rice has more vitamin A, which is necessary for maintaining eyes healthy, the risk of blindness has decreased globally (Shahbaz et al., 2020). Bioinformatics technologies are also essential for horticulture and agriculture from a climate change view. Certain cereal cultivars have been improved to thrive on poor soils and adapted to be resistant to drought and submersion (Zenda et al., 2021).

**Figure 4:**
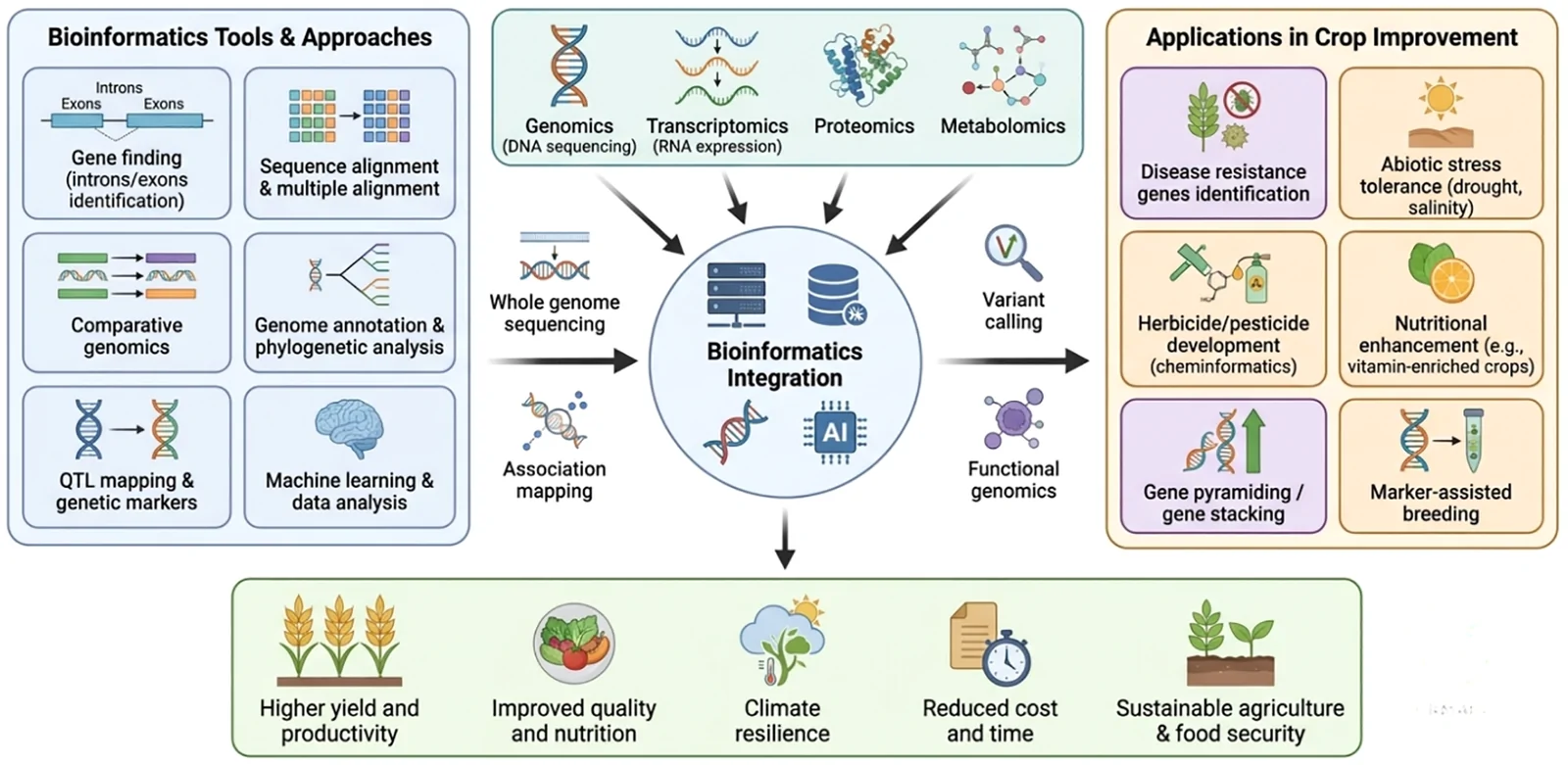
A comprehensive framework of bioinformatics integration in modern crop breeding.

A sustainable way to boost production and yield stability without increasing the usage of pesticides and fertilizers is to improve crops via breeding. Plant genome assembly issues brought on by polyploidy and numerous repetitive regions are being addressed with the use of third-generation sequencing technology (Hafeez et al., 2023). As a result, there are more high-quality crop reference genomes accessible. use further analysis like association mapping and variant calling that determine the genome’s breeding targets (Hu et al., 2018). Machine learning also helps in the identification of genomic areas of agronomic significance by enabling functional annotation of genomes and real-time high-throughput phenotyping of agronomic characteristics in the field and glasshouse (Mansoor et al., 2024). **Figure 4** presents the integration of multi-omics datasets through bioinformatics to support advanced crop improvement.

The schematic highlights the essential role of Bioinformatics Tools & Approaches ranging from genome annotation to machine learning in processing multi-omics data (genomics, transcriptomics, etc.). This integration enables high-precision Applications in Crop Improvement, including gene pyramiding and nutritional enhancement. The final output (bottom) demonstrates how this data-driven approach ensures sustainable agriculture by delivering high-yielding, climate-resilient crops with reduced developmental costs and time.

## 6 Bioinformatics in crop breeding: adapting to a changing climate

**Figure 5:**
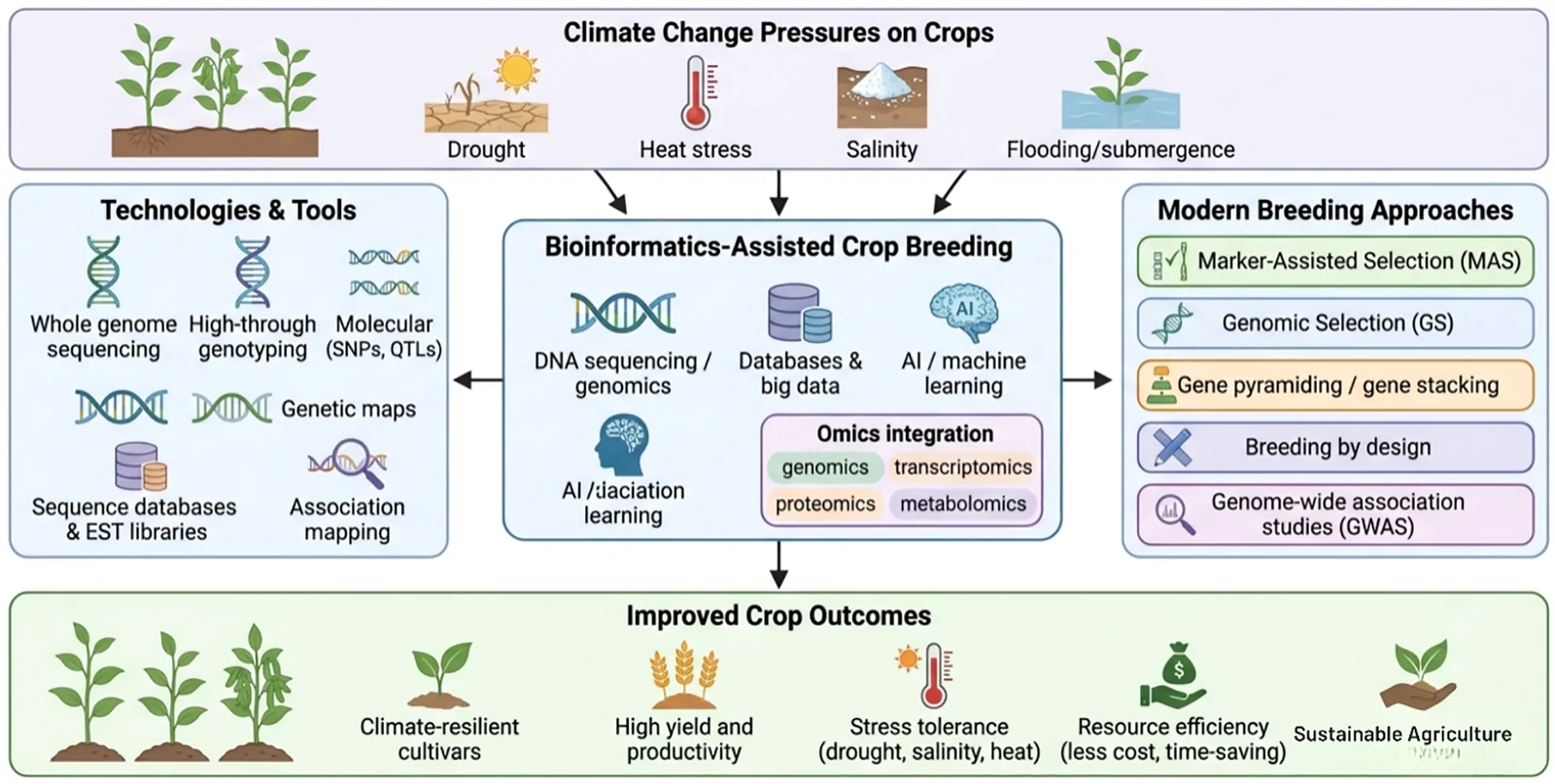
Bioinformatics-driven workflow for climate-resilient crop breeding.

The practice of “plant breeding is the art and science of changing the genetic structure of plants to produce desired characteristics”. Plant breeding is one of the many scientific fields in which bioinformatics has been used, and many of these methods and tools are under the omics category (Thriveni et al., 2024). Plant breeding has expanded in recent years with the creation and use of several techniques and instruments related to certain goals. Omics has enhanced the consistency and predictability of plant breeding programs, saving time and money on stress-tolerant cultivars (Saleem et al., 2025). The rapid advancement of genomics and its application to plant breeding began with the genome sequencing of crops, which was followed by several genome sequencing efforts of various plant species (Sun et al., 2022). A novel genomics-based plant breeding method is being developed by combining traditional breeding techniques with genomic tools and methods. Breeders will be able to identify genes linked to agronomic features, identify their location and function, and create genome-wide molecular markers with a completely assembled and well-annotated genome (Hu et al., 2018). The development of many databases and the replacement of traditional molecular marker technology with high-throughput DNA sequencing technologies are two of the most significant changes of bioinformatics approaches in plant breeding (Nadeem et al., 2018). Large collections of markers, high-throughput genotyping techniques, high-density genetic maps, and other genome-wide molecular tools that can be included in current breeding techniques are made available to breeders by these and other technological advancements. These sequence databases have developed into highly effective sequence resources for creating molecular markers across whole genomes because to the advancements in genome sequencing and large-scale EST analysis in a variety of species (Kumar et al., 2024). New plant breeding techniques, such as association mapping, marker-assisted selection, “breeding by design,” genomic selection, gene pyramiding, etc., are being produced by recent advancements in genomics (Singh and Singh, 2015). **Figure 5** shows a few of these molecular and genetic markers that have significantly improved plant breeding.

The schematic illustrates the strategic response to Climate Change Pressures (top) through a centralized Bioinformatics-Assisted Crop Breeding hub. By integrating diverse Technologies & Tools (left) such as whole-genome sequencing and molecular markers with Modern Breeding Approaches (right) like Genomic Selection and Gene Pyramiding, the system accelerates the development of elite varieties. This holistic approach leads to Improved Crop Outcomes (bottom), characterized by climate-resilient cultivars, enhanced resource efficiency, and sustainable agricultural productivity.

For decades, crop breeders have been aware of the complexity of many alleles.

However, the development of molecular markers has made it possible for breeding populations to exhibit genetic variety and other types of genetic organization (Amiteye, 2021). Numerous technologies for high-throughput genotyping have been developed and used for QTL cloning employing multiple segregation populations, marker-assisted selection, and genetic map creation. These genotyping technologies have also been applied to post-genome sequencing initiatives, including genotyping genetic resources, assessing population structure through accessions, and identifying genetic loci responsible for phenotypic changes in species through association studies (Sun et al., 2024).

MAS in plant breeding and a broader spectrum of study, including species identification and evolution, have benefited greatly from the use of molecular DNA markers (Song et al., 2023). Genetic markers developed to cover a large portion of the genome enable the investigation of genetic diversity in relation to natural variability in addition to identifying specific genes linked to complex characteristics through QTL analysis (Bidyananda et al., 2024). Many attempts have been made for different species, such as genome-wide rice (*Oryza sativa*), to create polymorphic markers from gathered sequence information. Datasets of DNA polymorphisms have been produced via the alignment of the genomes of indica and japonica rice (Hechanova et al., 2021). The most significant database EST (expressed sequence tag) is made up of ESTs extracted from various cDNAs. Sequence polymorphisms can also be found using large-scale EST datasets, particularly when assigning expressed genes to a genetic map (Sharma et al., 2022).

The Illumina GoldenGate Assay has been used to analyze genotypes of segregating populations to establish genetic maps assigning SNP (single nucleotide polymorphism) markers in crops. It enables the simultaneous study of up to 1536 SNPs in 96 samples (You et al., 2018). The high-throughput genotyping method known as Diversity Arrays Technology (DArT) was created using a microarray platform. These DArT markers have been employed in combination with traditional molecular markers to do association studies in a variety of crop species and/or create denser genetic maps (Deres and Feyissa, 2023). The Affymetrix Gene Chip Nucleotide polymorphisms as single-feature polymorphisms identified through the differential hybridization of Gene Chip probes in wheat and barley have been found using arrays (Kaur et al., 2021). Many scientists are attracted to the omics subcategories of transcriptomics, particularly in the field of plant breeding. The construction of enhanced contiguous crop genomes is the most effective use of third-generation sequencing for breeding (Mahmood et al., 2022) (**Table 6**).

**Table 6:** Molecular marker database and GWAS acceleration tools.

Databases	Description	Reference
GnpIS-Asso (association module of the GnpIS genomic/phenotypic database)	A general database for organizing and utilizing genetic association research on plants. It gives plant scientists and breeders the means to acquire association values between characteristics and markers from many association studies. Currently, GWAS on maize and tomatoes is carried out using this database.	(Ong et al., 2016)
BHIT (Bayesian High-order Interaction Toolkit)	The Bayesian high-order interaction toolkit (BHIT) identify high-order interactions in SNPs linked to case-control or quantitative traits by first constructing a Bayesian model on both continuous and discrete data. High-order interactions linked to phenotypes are efficiently detected by BHIT.	(Walakira et al., 2022)
Heap	SNP discovery tool for NGS data with particular reference to GWAS and finds more variations through the use of the knowledge whether or not the samples are assumed to be inbred homozygous.	(Kobayashi et al., 2017)
BioGPU (GPU-accelerated GWAS tool)	When compared to mixed linear model approaches, BioGPU, a high-performance computing tool for GWAS, substantially reduces false positives brought on by population structure and uneven relatedness among individuals and increases statistical power.	(Ong et al., 2016)
GrainGenes	It is a recognized website for Triticeae genomics that offers linkage map information and genetic markers for wheat, barley, rye, and oats.	(Yao et al., 2022)
Triticeae mapped EST database	(TriMEDB, Triticeae Mapped EST DataBase) offers details on mapped cDNA markers associated with barley and their wheat equivalents.	(Mochida et al., 2008)
MaizeGDB	A search engine for maize sequencing data and ESTs, AFLPs, and RAPD probes. For a variety of legumes, such as peanuts, soy beans, alfalfa, and common beans, the Legume Information System (LIS) offers markers including SNP, SSR, RFLP, and RAPDs.	(Harper et al., 2016)
SoyBase database	Hosts genetic and genomic information on soy beans. The markers consist of SNP, SSR, RFLP, RAPD, and AFLP.	(Grant and Nelson, 2017)
The cotton microsatellite database (CMD)	A web-based relational database that is curated and connected, giving centralized access to cotton SSRs that are available to the public. For nine significant cotton SSR experiments, CMD includes publication, sequence, primer, mapping, and homology data, totaling 5484 SSR markers.	(Savadi et al., 2023)
SSR primer 2	It enables the simultaneous creation of PCR primers for SSR amplification and the real-time identification of SSRs within supplied DNA sequences. Brassica and strawberries have shown this system’s effectiveness.	(Azizi et al., 2021)
MoccaDB	An integrated database for research on variety, comparison, and function in the Rubiaceae family, which includes coffee. It makes markers like SSR, SNP, and RFLP easily accessible.	(Plechakova et al., 2009)
Sol genomics network database	Chromosomes, genes, phenotypes, and varieties in the Solanaceae and their near relatives are among the data kinds. For tomatoes, potatoes, peppers, and tobacco, SGN has over 20 genetic and physical maps with thousands of markers. The database contains genetic marker types such as SNP, SSR, AFLP, PCR, and RFLP.	(Fernandez-Pozo et al., 2015)
ICRISAT	Over 2800 chickpea ESTs from a library created by subtractive suppressive hybridization (SSH) are accessible through a chickpea (Cicer arietinum L) root EST database hosted at ICRISAT.	(Lai et al., 2014)

Furthermore, it will become more feasible to link candidate genes found in model species with associated loci in crop plants as the accuracy of genetic maps in various crops rises and the molecular basis for particular traits or responses is better understood (Chavhan et al., 2024). It will be feasible to freely interact across genomes in terms of gene sequence, putative function, and genetic map position with the use of suitable relational databases. The difference between molecular genetics and breeding will disappear once such methods are put into use. Breeders frequently utilize computer models as a toolbox to formulate prediction hypotheses to develop phenotypes of interest from complicated allele combinations. They then construct such combinations by scoring huge populations for a large number of genetic markers (Washburn et al., 2020).

## 7 Bioinformatics platforms and applications in crop breeding

Phenotypic selection and crossing cycles have long been the foundation of crop breeding, which use genetic recombination to produce better genotypes. When sequences of genomes are accessible, all genes and genetic variations that influence agricultural characteristics may be determined, and modifications made during the breeding process can be evaluated at the genotype level (Singh et al., 2022a). In every aspect of crop breeding, including genome-wide association studies (GWAS) and quantitative trait loci (QTL) mapping, where genomic sequencing of crop populations can enable gene-level resolution of agronomic variation, the availability of readily available genomic data for breeders is becoming more and more crucial (Desaint et al., 2023). Even the identification of genetic diversity in crop species, which are used to create climate-resilient variants, has been made possible by advancements in genomics-based breeding (Mondal et al., 2023).

Nowadays, GWAS (phylogenomics, comparative genomic analysis, evolutionary analysis, and genome-wide association research) is a useful method for investigating allelic variation in a wider range for greater phenotypic variety and QTL mapping resolution (Sahito et al., 2024). The limitations of current traditional crop breeding techniques, such as a biparental cross-mapping approach for genetic dissections of the agronomically significant characteristics, are addressed using GWAS (Khan et al., 2021). In plant breeding, GWAS is an effective method for determining allelic variation in candidate genes that address quantitative and complex traits as well as phenotypic diversity in trait-associated loci (Altaf et al., 2024). *Arabidopsis thaliana* has been effectively studied using GWAS, with over 1300 different accessions genotyped for 250,000 SNP traits. Yield, stress tolerance, morphology, and nutritional quality were all determined to be influenced by a small number of rice genes (Atwell et al., 2010). GWAS has been widely utilized to analyze complex features in a number of other important crops, such as soybeans and maize. A number of bioinformatics techniques have been presented as GWAS acceleration tools (Sahito et al., 2024).

Genomic advancements have the potential to rapidly speed up crop plant breeding based on genetics. However, connecting genetic data to climate-related agricultural features for breeding purposes is still very difficult and will need knowledge and abilities (Marsh et al., 2021; Mujahid et al., 2025). Combining genetics and bioinformatics assist in sustaining food security in the context of climate change by accelerating the generation of crops that are climate-ready. The extensive breeding information acquired over the past few decades will improve the ability to clarify gene function in model organisms and be directly related to fundamental plant biology (Razzaq et al., 2021). Both the enhancement of elements that typically restrict agronomic performance (input traits) and the modification of the quantity and types of materials that crops generate (output characteristics) are expected to result in phenotypes of economic interest (Singh et al., 2024). Examples include: biotic stress tolerance, abiotic stress tolerance, enhancing nutrient use efficiency, manipulation of the size, shape, quantity, and location of organs, as well as the timing of senescence and development, metabolite partitioning is the process of shifting carbon flow into new channels or rerouting it among existing ones.

It will be able to freely link genes across genomes based on gene sequence, suspected function, or genetic map position with the use of appropriate relational databases. The difference between molecular genetics and breeding will disappear once such techniques are implemented into practice (Yu et al., 2021). To develop phenotypes of interest from complex allele combinations, breeders often use computer models to formulate predictive hypotheses. These hypotheses are subsequently constructed by scoring large populations for a large number of genetic markers (Chavhan et al., 2024).

### 7.1 Applications in fruit crop genomics and breeding

Building on the general framework established above, fruit crop breeding illustrates a domain-specific application of these same principles rather than a separate set of goals. Advances in fruit plant genomics over the past three decades have expanded understanding of the molecular basis of fruit quality and production traits, abiotic stress tolerance, and pathogen resistance (Buch et al., 2023; Sarfraz et al., 2025b). Gene expression analysis has been particularly informative for understanding how fruit trees respond to their physical environment and management practices, feeding directly into breeding-decision support systems that combine predicted fruit-quality and plant-health assessments (Bhuiyan et al., 2025; Singh et al., 2022b). As with the field-crop applications discussed earlier, the resulting sequencing, marker, map, mutation, and functional-annotation data must be processed, stored, and shared with the broader scientific community; the priorities specific to fruit plant bioinformatics are therefore to integrate phenotypes, genomes, and bioinformatics tools within public and commercial breeding pipelines, to supply consistent gene, protein, and phenotype annotation, and to clarify comparative linkages between fruit species and other organisms.

## 8 Limitations, challenges, and future directions

Despite the substantial progress reviewed above, the routine application of bioinformatics-driven crop improvement still faces several practical, biological, and regulatory constraints that must be acknowledged if these methods are to be deployed equitably and effectively across breeding programs worldwide.

(a) Computational cost and infrastructure barriers: High-throughput sequencing, genome assembly, and genomic prediction pipelines require substantial computational infrastructure, data storage, and bioinformatics expertise. These resources remain unevenly distributed, and breeding programs in low- and middle-income countries, where genetic gains are often most urgently needed for food security, frequently lack the computing capacity, high-speed connectivity, and trained personnel to implement genomic selection or large-scale multi-omics analysis at the same scale as well-resourced programs, creating a widening technological gap in global crop improvement capacity (Santantonio et al., 2020).
(b) Genotype-by-environment (G×E) interactions: Genomic prediction models are typically trained in a limited set of environments, yet the trait performance they are meant to predict is strongly influenced by G×E interactions (Crossa et al., 2021). Prediction accuracy can drop substantially when models are applied to environments, management practices, or climate conditions not represented in the training population, which is a particular concern given that climate change is shifting the very environmental conditions breeding programs must target. Incorporating environmental covariates and multi-environment trial data into prediction models is an active area of methodological development but adds considerable complexity and data requirements (Crossa et al., 2021).
(c) Ethical, regulatory, and biosafety considerations for CRISPR-edited crops: Regulatory frameworks for genome-edited crops differ markedly between jurisdictions, ranging from treatment equivalent to conventional breeding (where no new DNA is introduced) to full regulation under genetically modified organism (GMO) legislation (Buchholzer and Frommer, 2023). This regulatory heterogeneity affects which CRISPR-edited varieties can be commercialized in a given market, creates uncertainty for breeding programs planning long-term projects, and can slow the translation of promising edits into farmer-accessible varieties. Public acceptance and biosafety assessment, particularly for edits involving multiplexed or heritable germline changes, remain important considerations alongside the technical feasibility of gene editing.
(d) Data privacy and intellectual property: Genomic and phenotypic databases increasingly aggregate proprietary breeding material alongside publicly available germplasm data. Questions of data ownership, benefit-sharing with the (often smallholder) farmers and communities who originally maintained genetic resources, and intellectual property claims over identified genes and markers, remain incompletely resolved, and inconsistent data-sharing policies across databases and countries can restrict the pooling of genomic data needed to improve genomic prediction accuracy, especially for underfunded orphan-crop programs (Spindel and McCouch, 2016).
(e) The gap between omics discovery and field deployment: A large proportion of candidate genes, markers, and QTLs identified through GWAS, transcriptomics, or other omics approaches are never functionally validated or advanced into elite breeding lines (Thomson et al., 2022). Bridging this gap requires sustained investment in functional validation (e.g., through gene editing or transgenic confirmation), multi-location and multi-year field trials, and close collaboration between bioinformaticians, molecular biologists, and field breeders, none of which is guaranteed by discovery-stage funding alone. Strengthening the pipeline from in silico discovery to on-farm cultivar release, rather than continuing to expand the discovery stage in isolation, is likely to be one of the most important determinants of whether bioinformatics-driven crop improvement translates into realized gains in global food security.

## 9 Conclusion

The field of crop improvement has undergone a revolution through the combination of bioinformatics and molecular breeding, offering effective tools and techniques to create crop varieties that are stronger, more productive, and more nutritious. These methods have made it possible to identify novel genetic markers and candidate genes for important agronomic traits, as well as to design and implement effective breeding strategies such as marker-assisted selection, genomic selection, and genome editing, using the advancements in genomic sequencing, data management, and analytical tools. The use of these strategies has produced novel crop types that satisfy the various demands and interests of farmers and consumers, as well as notable gains in crop production, quality, and resistance to biotic and abiotic challenges.
